# 

**Published:** 1836-07-01

**Authors:** 


### CONTENTS

OF THE

### MEDICO-CHIRURGICAL REVIEW,

No. XLIX. JULY 1, 1836,

[BEING No. IX. OF A DECENNIAL SERIES.]

### REVIEWS.

I.

An Essay on Laryngismus Stridulus, or Croup-like Inspiration of Infants. By Hugh Ley, M.D... .. .. .. .. .. .. .. .. .. .. .. .. .. .. 1

II.

Cases and Observations illustrative of Renal Disease, accompanied with the Secretion of Albuminous Urine. By Dr. Bright .. .. .. .. .. .. .. .. .. .. .. 23

III.

Principles and Practice of Obstetric Medicine. By Dr. Davis.—(Malposition of Uterus) 36

IV.

MIDWFFERY.

1. A Practical Treatise on Midwifery. By Robert Collins, M.D. .. .. .. ..
2. Practical Observations on various Subjects relating to Midwifery. By James Hamilton, M.D... .. .. .. .. .. . .. .. .. .. .. .. .. .. ..
3. Observations on the Propriety of inducing Premature Labour in Pregnancy, complicated with Tumor. By Dr. Ashwell .. .. .. .. .. .. .. .. .. .. .. 38

V.

A Treatise on the Chemical, Medicinal, and Physiological Properties of Creosote, illustrated by Experiments, &c. By John Rose Cormack .. .. .. .. .. .. 69

VI.

Medical Commentaries on Puerperal Fever, Vermination, and Water in the Head. By John Alexander, M.D. .. .. .. .. .. .. .. .. .. .. .. .. 72

VII.

Lectures on Subjects connected with Clinical Medicine. By Dr. Latham .. .. .. 75

VIII.

Nouvelles Recherches sur le Rheumatisme Articulaire Aigu, &c.—New Researches respecting Acute Articular Rheumatism in general, and especially on the Law of Coincidence between that Disease and Pericarditis, &c. By J. Bouillaud, M.D. 82

IX.

Lectures on the Nervous System and its Diseases. By Marshall Hall, M.D.—(On Laryngismus Stridulus) .. .. .. .. .. .. .. .. .. .. .. .. .. 92

X.

BRIDGEWATER TREATISES.

Chemistry, Meteorology, and the Functions of Digestion, considered with reference to Natural Theology. By William Prout, M.D. .. .. .. .. .. .. .. .. 95
1. Organic Bodies .. .. .. .. . .. .. .. .. .. .. .. .. .. .. .. 96
2. Nutrition of Plants .. .. .. .. .. .. .. .. .. .. .. .. .. .. 101
3. Nutrition of Animals.. .. .. .. .. .. .. .. .. .. .. .. .. 102

XI.

A Dictionary of Terms employed by the French in Anatomy, Physiology, Pathology, Practical Medicine, Surgery, Midwifery, Pharmacy, Medical Zoology, Botany, and Chemistry; with their Derivations from the Greek and Latin; their Synonyms in the Greek, Latin, French, German, and English; and Illustrations in the different Languages. By SHIRLEY PALMER, M.D. Part II.. .. .. .. .. .. 117

XII.

PATHOLOGICAL ANATOMY.

Illustrations of the Elementary Forms of Disease. By Dr. CARSWELL.—(Atrophy) .. 120

XIII.

The Transactions of the Provincial Medical and Surgical Association .. .. .. .. .. 130

1. An Account of the Autumnal Fevers, Epidemic Scarlatina, and Small-pox, which prevailed at Amsterdam during the Year 1834. By C. J. NIEUWENHUYS, M.D.. .. .. .. .. .. .. .. .. .. .. .. .. .. .. 131
2. Memoir upon the Method of securely closing Moist Anatomical Preparations, preserved in Spirit. By JOHN GREEN CROSSE, Esq. Surgeon to the Norfolk and Norwich Hospital, &c. .. .. .. .. .. .. .. .. .. .. .. .. 136
3. Mr. KNOWLES' Case of Deformity of the Pelvis .. .. .. .. .. .. .. 143

### PERISCOPE.

PART I.

### Spirit of the English Periodicals, and Notices of English Medical Literature.

1. On the Modus Operandi of Medicines. By James Johnson, M.D... .. .. .. 145
2. Quackery, its Danger, Irrationality, &c. .. .. .. .. .. .. . .. .. .. .. 149
3. On the Medicinal Effects of Carbonate of Soda in large Doses. By Mr. Neville .. 150
4. The Philosophy of Decapitation .. .. .. .. .. .. .. .. .. .. .. .. .. 151
5. On Spontaneous Rupture of the Heart. By Dr. Hallowell .. .. .. .. .. .. 152
6. Remarkable Case of Occlusion of the Cystic Duct—Lining Membrane of the Gall-Bladder changed into a Pus-secreting Surface—Gall-bladder enormously enlarged, and filled with Pus and Gall-stones. By the Editors .. .. .. .. .. .. 155
7. Paralysis of the Tongue and Muscles of Deglutition, without Apoplexy, or any Diminution of the Sensorial Powers. Appearances on Dissection. By James Johnson, M.D. .. .. .. .. .. .. .. .. .. .. .. .. .. .. .. .. .. 156
8. The Advantages of New Remedies .. .. .. .. .. .. .. .. .. .. .. .. 158
   Colchicum in Tetanus. By Dr. Smith, of America .. .. .. .. .. .. .. 159
9. German Contributions to Practical Medicine and Surgery. By Dr. Thacker.. .. 162
   1. Polypus Uteri, with Unusual Symptoms. By Dr. Buck.. .. .. .. .. .. 162
   2. Vaginal Hernia of the Bladder, of most difficult Diagnosis .. .. .. .. 164
   3. Compound Fracture of Skull .. .. .. .. .. .. .. .. .. .. .. .. .. 167
   4. Hydrocephalus Chronicus Externus.. .. .. .. .. .. . .. .. .. .. 168
10. Singular Case of Paralysis Crescens ab imo. By D. Johnson .. .. .. .. .. .. 169
11. Medical Statistics—
   1. Observations on the Mortality in Philadelphia under the Age of Puberty, &c.&c. By G. Emerson, M.D... .. .. .. .. .. .. .. .. .. .. .. .. .. 169
   2. Comparative Mortality of Male and Female Life . .. .. .. .. .. .. 172

3. Increased Value of Life in England—(Mr. Rickman) .. .. .. .. .. .. 174
4. Mortality of Medical Men .. .. .. .. .. .. .. .. .. .. .. .. 174
5. The Married and Single compared .. .. .. .. .. .. .. .. .. 175
12. On Periostitis of the Orbit. By Mr. Hamilton .. .. .. .. .. .. .. 176
13. On the Use of the Cochlea in the Organ of Hearing. By Dr. Weber .. .. .. 177
14. Professor Harrison on the Crystals observed on the Human Peritoneum .. .. 180
15. Maltreatment of a fractured Leg. By Messrs. Boyer and Deschamp .. .. .. 181
16. Excision of the Cyst of a Strangulated Umbilical Hernia .. .. .. .. .. .. .. 182
17. On the Sounds of Substernal Aneurisms. By Dr. Henderson .. .. .. .. 183
18. Case of Recovery from Dislocation and Fracture of the Spine. By Dr. Dorrance.. 185
19. Successful Amputation during the Progress of Traumatic Gangrene. By Mr. Eve, 187
20. Professor Mueller's Account of the Arteries that produce Erection of the Penis .. 188
21. Mr. Ure on the Chloride of Zinc, as a Remedy for Cancer .. .. .. .. .. 190
22. Some Cases of Disease of the Kidney, in which the Symptoms were referred to the Bladder. By Henry James Johnson, Esq. .. .. .. .. .. .. .. .. .. 193
23. Strictures on Dr. Ryan's Translation of a " New Practical Formulary of Hospitals, &c." By T. P. .. .. .. .. .. .. .. .. .. .. .. .. .. .. 199

### Notices of New Works.

24. The Cyclopædia of Anatomy and Physiology. Edited by R. Todd, M.B. .. .. 202
25. Practical Anatomy of the Nerves and Vessels supplying the Head, Neck, and Chest, &c. By Ed. Cock, Esq. .. .. .. .. .. .. .. .. .. .. .. .. .. 205
26. Compendium of the Ligaments, &c. By A. M'Nab, Jun. .. . .. .. .. 206
27. The Surgeon's Practical Guide, &c. By Thomas Cutler, M.D. .. .. .. .. 206
28. A Series of Anatomical Plates. By Jones Quain, M.D. Fas. 30, 31, 32 .. .. 207
29. The Clinique Medicale; or Reports of Medical Cases. By G. Andral, &c. Translated by Dr. Spillan. Part IV. Diseases of the Abdomen .. .. .. .. .. 207
30. Manuel de Medicine Operatoire, &c. Par M. Malgaigne, M.D. .. .. . .. 208

PART II.

### Spirit of the Foreign Periodicals, &c.

1. Hints respecting the Changes which the Urine undergoes in Disease, and the Therapeutic Treatment, &c.—(Eclectic) .. .. .. .. .. .. .. .. .. .. .. .. 208
2. Detection of Sulphate of Quinine in Urine. By M. Piorry .. .. .. .. .. .. 215
3. Observations on some of the Forms of Monomania. By M. Henke .. .. .. 216
4. On the State of Medical Doctrine in France .. .. .. .. .. .. .. .. .. 223
5. On the Endermic Use of Quinine in Ague. By Dr. Chomel .. .. .. .. .. 228
6. On the Endermic Use of Digitalis in Affections of the Heart .. .. .. .. .. 229
7. Baron Alibert on the Cheloid Tumour of the Skin .. .. .. .. .. .. .. .. 231
8. M. St. Hilaire on Albinism and Melanism .. .. .. .. .. .. .. . .. .. 234
9. On the Odoriferous Exhalation from the Skin. By M. Rayer .. .. .. .. 236
10. New Medical Journal set up in India .. .. .. .. .. .. .. .. .. .. .. .. 238
11. Sketch of Mr. Liston, written on the Banks of the Ganges .. .. .. .. .. 238
12. Remarkable Case of Hemiplegia.. .. .. .. .. .. .. .. .. .. .. .. .. 239

PART III.

### Clinical Review, and Hospital Reports.

St. Thomas's Hospital Reports.

1. Dr. Roots on Porrigo Lupinosa .. .. .. .. .. .. .. .. .. .. .. .. .. 241
2. Hysterical Paralysis (with Commentaries) .. .. .. .. .. .. .. .. .. 242

3. Compound Dislocation of the Clavicle .. .. .. .. .. .. .. .. .. .. .. 246
4. Chronic Dysentery .. .. .. .. .. .. .. .. .. .. .. .. .. .. 246
5. Mr. Tyrrell on Diseases of the Joints .. .. .. .. .. .. .. .. .. .. 248
6. Dr. Williams on the Treatment of Idiopathic Erysipelas by Wine .. .. .. .. 251
7. Diffused Tuberculation.. .. .. .. .. .. .. .. .. .. .. .. .. .. 256

QUEEN'S COUNTY INFIRMARY

8. Rapid Recovery of Lunatics.. .. .. .. .. .. .. .. .. .. .. .. .. 259

9. Anomalous Hysterical Affections.—Dr. Jacob .. .. .. .. .. .. .. .. .. 259

10. Mode of treating Fracture of Cervix Femoris .. .. .. .. .. .. .. .. .. 260

11. Swallowing of Pins .. .. .. .. .. .. .. .. .. .. .. .. .. .. 260

12. Remarks on Catheterism in Retention of Urine .. .. .. .. .. .. .. .. 261

13. Malignant Hæmorrhagic Tumours .. .. .. .. .. .. .. .. .. .. .. .. 262

GLASGOW ROYAL INFIRMARY.

14. On Phlebitis, particularly as connected with Secondary Abscesses. By James Douglas, A.M. with numerous Cases.. .. .. .. .. .. .. .. .. .. 263

### Miscellanies.

1. On the prone Position in Affections of the Spine, Hips, &c. By Mr. Verral .. 275
2. On Bleeding in the Cold Stage of Intermittents.. .. .. .. .. .. .. .. .. 278
3. On reporting from Medical Societies .. .. .. .. .. .. .. .. .. .. .. 279
4. The Dublin Journal .. .. .. .. .. .. .. .. .. .. .. .. .. .. 279
5. Memoir of Sir William Blizzard .. .. .. .. .. .. .. .. .. .. .. .. 280
6. Mesenteric Disease. Peritoneal Accretion. By Mr. Green .. .. .. .. .. .. 283
7. Mr. Wallace's instances of Want of Sagittal Suture in Negroes .. .. .. .. 285
8. Eastern Provincial Association .. .. .. .. .. .. .. .. .. .. .. .. 286
9. Barry O'Meara, Esq. .. .. .. .. .. .. .. .. .. .. .. .. .. .. 286
BIBLIOGRAPHICAL RECORD .. .. .. .. .. .. .. .. .. .. .. .. .. .. 287

EXTRA LIMITES.

Notes on the Epidemic Yellow Fever at Antigua. By Dr. Furlong .. .. .. .. 289

